# ENHANCING DIGNITY IN DEMENTIA CARE IN BRAZIL: A CAREGIVER REMOTE 4MS FRAMEWORK APPLICATION FOR MOBILITY SOLUTIONS

**DOI:** 10.1093/geroni/igae098.0299

**Published:** 2024-12-31

**Authors:** Patricia Heyn

**Affiliations:** Marymount University, Fairfax Station, Virginia, United States

## Abstract

This abstract describes a case study based on the personal experience of an Alzheimer’s researcher who unexpectedly turned into a caregiver and crafted a novel care paradigm for her father with dementia living in a nursing home in Brazil. Confronting Brazil’s traditional care models, which often exacerbate frailty by confining older adults to wheelchairs, she championed the Age-Friendly Health Systems movement and its core 4Ms framework—Medication, Mobility, Mentation, and What Matters the Most. This personalized approach, advocating for autonomy rather than restraint, sparked a systemic shift toward recognizing and nurturing the functional capabilities of older adults with dementia. Implementing this framework virtually, we are capable of training a caregiver remotely through WhatsApp to deliver tailored physical and cognitive exercises for a physically restrained person with dementia, demonstrating the feasibility of technology-facilitated caregiving. This intervention led to significant functional improvements: the transition from wheelchair to walker, then to cane, and finally, to unassisted ambulation, and improved strength and walking speed within a year. Highlighting this success, the presentation will discuss the transformative influence of the Aging Friendly 4Ms framework on person-centered geriatric care in an international context and the potent role of technology in maintaining family engagement and oversight in caregiving quality from afar. This case underscores the necessity of personalized care that regenerates and maintains active mobility, ultimately preserving the dignity, independence, and quality of life for older adults with dementia in underdeveloped institutional settings.

